# Height and weight reference charts for Brazilians with intellectual disabilities aged 7–17

**DOI:** 10.1016/j.jped.2024.11.004

**Published:** 2024-12-04

**Authors:** Adriana Nascimento de Souza, Fabio Bertapelli, Gil Guerra-Junior

**Affiliations:** a Universidade Estadual de Campinas (UNICAMP), Faculdade de Ciências Médicas (FCM), Centro de Investigação em Pediatria (CIPED), Laboratório de Crescimento e Desenvolvimento (LabCreD), Campinas, SP, Brazil; b Universidade Estadual de Campinas (UNICAMP), Faculdade de Ciências Médicas (FCM), Departamento de Pediatria, Divisão de Endocrinologia Pediátrica, Campinas, SP, Brazil

**Keywords:** Body weight, Body height, Growth and development, Growth disorders, Children with disabilities

## Abstract

**Objective:**

It was to develop smoothed height and weight percentiles for boys and girls with IDs between 7 and 17 years old.

**Methods:**

The sample consisted of 1,047 young people (645 boys and 402 girls; 7–17 years old) with ID. A total of 4,059 measurements (height: *n* = 2,041; weight: *n* = 2,018) were retrospectively obtained from the period between 2013 and 2018. Smoothed height and weight percentiles were developed using the LMS method. Local and global diagnosis of percentiles were evaluated with Q statistics and detrended Q-Q plots.

**Results:**

Percentiles (5, 10, 25, 50, 75, 90, and 95) for height-to-age and weight-to-age were developed with satisfactory modeling in boys and girls between 7 and 17 years old. Boys showed a linear trend in height up to 11 or 12 years old, an increment from 13 to 15 years old and a deceleration from 15 or 16 years old. For the girls, height was linear between the ages of 7 and 11, followed by a deceleration from the age of 12 and without substantial changes from the ages of 15 to 17. Regarding weight, girls showed a linear trend of weight gain until the age of 13 and deceleration at the age of 14 or 15. Boys, however, showed a linear tendency to gain weight from 7 to 17 years old.

**Conclusion:**

The height and weight percentiles developed in this study can help monitor the growth of young people with ID.

## Introduction

Intellectual Disability (ID) is a condition characterized by limitations in cognitive functioning and adaptive behavior, manifesting before the age of 22, bringing several losses to the individual's full participation in society.^1^ ID occurs in around 1 to 3 % of the population, with a higher occurrence in young males, in low-income countries.^2^ According to data from the 2010 Demographic Census, there are at least 2.6 million (1.4 %) people with ID in Brazil.^3^ More recent data indicate that the occurrence of children with ID is equivalent to 0.85 %.^4^

Studies indicate a greater risk for physical and mental disorders, premature mortality and potentially preventable deaths among individuals with ID.^5^, ^6^, ^7^ In the population of children with ID, evidence suggests a greater risk for growth disorders and overweight.^8^ More specifically, studies suggest that young people with ID have smaller height and greater weight compared to young people without ID.^9^^,^^10^ However, there is a lack of data regarding growth patterns in the population of children with ID.

The absence of specific reference values ​​has resulted in the development of national references for growth in the population with ID. However, the charts were developed for children with ID associated with genetic conditions, such as Down,^11^ Willians,^12^^,^^13^ Rett,^14^ Rubinstein-Taybi,^15^ Prader-Willi^16^ syndromes, among others.

Genetic conditions are associated with lower height and weight compared to the general population of young people with ID,^10^ which may result in overestimated growth values ​​for young people with ID without associated genetic conditions. The development of height and weight percentiles should help in the construction of specific growth trajectories in the population with ID in order to guide public policies to prevent growth disorders in young people with ID.

Growth is a fundamental indicator of health and social development.^17^ To this end, growth charts have been developed and used to monitor height and weight in children.^18^, ^19^, ^20^ The charts are essential components for evaluating the general health status of the population. However, reference values used for children in the general population may be inadequate for monitoring the growth of young people with ID.^21^

The aim of the present study was to develop smoothed height-to-age and weight-to-age percentiles for Brazilian children and adolescents with ID, aged between 7 and 17 years.

## Methods

### Sample

It contains individuals with ID of both sexes, aged between 7 and 17 years, from 45 cities in the State of Sao Paulo, Brazil. Young people with recognized genetic conditions, cerebral palsy, or severe physical disabilities were excluded from the study. The study was approved by the Research Ethics Committee (assessment n° 3.419.135) for the use of clinical records.

### APAE health database – FEAPAES

Demographic data, height and weight were obtained from the APAE Health program – Federation of APAES of the State of Sao Paulo (FEAPAES-SP). FEAPAES is a non-profit organization dedicated to the promotion, dissemination and development of educational and health projects at specialized centers (APAES) for people with ID. The APAE Health program is a FEAPAES initiative and aims to obtain reliable and comparable data on nutritional conditions and cardiovascular profiles of samples from individuals with ID. FEAPAES coordinated data collection from APAES from the period between 2013 and 2018. First, in 2013, a pilot collection was carried out in two APAES, where a questionnaire was filled out, containing the following information: date of birth, age and sex. Next, APAES measured the height and weight of children and adolescents with ID. After the pilot study, all evaluators underwent training sessions, with professionals linked to APAES (Physical Education teachers, Nutritionists, Nurses, Physiotherapists). Data collection was carried out in 45 APAES. Height and weight data were obtained following standardized measurement protocols from APAE Health – FEAPAES. Height and weight were obtained using stadiometers and scales with precision of 0.1 cm and 0.1 kg, respectively. Measurements were taken with participants standing, wearing light clothing and without shoes.

### Statistical analysis

The data was subjected to the cleaning process. Height and weight data were excluded under the following conditions: 1) height and weight data with duplicate ages (height: *n* = 332; weight: *n* = 315) and, 2) loss of height in the longitudinal sample (*n* = 61).

The LMS method was used to develop percentiles (5, 10, 25, 50, 75, 90 and 95),^22^ using the LMSchartmaker Pro software.^23^ Values ​​of L, M and S represent the adjusted Box-Cox power, median and coefficient of variation, respectively. The LMS fits skewed data, using a Box-Cox normal distribution. The modeling diagnosis was based on Q statistics values ​​for L, M and S between −2 and +2 and detrended Q-Q plots (worm plots) using LMSchartmaker Pro. The diagnosis is based on choosing the best set of degrees of freedom for cubic splines and goodness-of-fit. The model was built with the “age original” option for age (i.e., without rescaling or transformations). The initial equivalent degrees of freedom (i.e., edf) for L, M, and S were 3, 5, and 3, respectively. The model checking was carried out following the recommendations of LMSchartmaker Pro: the authors initially sought to optimize the M, S and L chart, increasing or decreasing the edf by 1 unit. The height and weight percentiles of male individuals presented satisfactory diagnoses (Q statistics between −2 and +2), using initial edf (*L* = 3, *M* = 5, *S* = 3). Modeling for female weight percentiles resulted in the extrapolation of Q statistics values; the edf change in the M curve did not improve the modeling, requiring a decrease in the S curve by 1 unit (*L* = 3, *M* = 5, *S* = 2), which resulted in Q statistics within −2 and +2. To model height percentiles, the M curve was increased by 1 unit (*L* = 3, *M* = 6, *S* = 3). Worm plots for height and weight resulted in a linear trend within the recommendations of van Buuren and Fredriks.^24^ The modeling results can be seen in Supplementary Fig. 2 in the supplementary materials. L, M and S values ​​and smoothed height and weight percentiles were exported from LMSchartmaker to an Excel spreadsheet to create height and weight tables and charts. Frequency, mean, and standard deviation was calculated using SPSS, version 22 (IBM, Armonk, NY).

## Results

### Participants

The sample consisted of a total of 1047 children and adolescents (male: *n* = 645; 61.6 %; female: *n* = 402; 38.4 %) with ID, aged between 7 and 17 years. Participants provided 2041 height data (male = 1249; female: *n* = 792) and 2018 wt data (male: *n* = 1228; female: *n* = 790). The percentages of participants who presented one or more measurements were: 1 measurement (male: 57.4 %; female: 55.9 %); 2–3 measurements (male: 28.4 %; female: 29.6 %); and 4–6 measures (male: 14.2 %; female: 14.6 %). The number of height and weight measurements by sex and age are presented in Table 1, Table 2.

**Table 1:** Average, standard deviation (SD), L, M, S, and smoothed height percentiles in male and female children and adolescents with intellectual disabilities aged between 7 and 17 years.

		Male (height, cm)											
Age (years)	n	Average	SD	L	M	S	P5	P10	P25	P50	P75	P90	P95
7	52	125.32	7.15	−0.3649	125.3823	0.0598	113.83	116.25	120.46	125.38	130.58	135.52	138.60
8	64	131.68	7.67	0.0358	130.9153	0.0609	118.42	121.08	125.65	130.92	136.40	141.52	144.67
9	96	136.03	8.96	0.5009	136.2581	0.0618	122.76	125.68	130.64	136.26	142.00	147.26	150.46
10	97	142.54	8.90	1.0716	141.4928	0.0625	126.90	130.13	135.52	141.49	147.44	152.79	155.98
11	123	145.28	8.70	1.6902	146.7092	0.0628	130.95	134.54	140.40	146.71	152.84	158.22	161.37
12	116	151.77	9.91	2.2332	152.4456	0.0629	135.52	139.47	145.80	152.45	158.76	164.18	167.32
13	134	157.84	10.40	2.5947	158.1908	0.0626	140.35	144.59	151.28	158.19	164.65	170.14	173.30
14	159	162.84	11.31	2.7130	162.9969	0.0615	144.81	149.16	155.97	163.00	169.54	175.07	178.25
15	141	165.89	9.51	2.6255	166.2806	0.0599	148.36	152.62	159.33	166.28	172.79	178.33	181.51
16	128	168.61	9.13	2.4396	168.2967	0.0581	150.95	155.02	161.51	168.30	174.71	180.21	183.39
17	139	168.42	9.82	2.2561	169.4808	0.0563	152.76	156.65	162.89	169.48	175.77	181.20	184.35

		Female (height, cm)											
Age (years)	n	Average	SD	L	M	S	P5	P10	P25	P50	P75	P90	P95

7	21	123.71	8.06	−0.3361	123.2524	0.0685	110.34	113.03	117.73	123.25	129.13	134.75	138.26
8	44	129.09	9.18	0.0159	129.0198	0.0656	115.82	118.61	123.44	129.02	134.85	140.32	143.70
9	47	135.33	9.54	0.3377	134.8608	0.0626	121.45	124.33	129.25	134.86	140.63	145.96	149.22
10	63	140.19	8.07	0.5617	140.6397	0.0596	127.15	130.08	135.04	140.64	146.34	151.56	154.72
11	69	146.72	7.41	0.6872	146.2879	0.0570	132.77	135.72	140.70	146.29	151.95	157.10	160.21
12	82	151.05	8.43	0.7742	150.8989	0.0551	137.37	140.33	145.32	150.90	156.53	161.63	164.71
13	72	154.50	8.31	0.9372	154.0020	0.0536	140.45	143.44	148.44	154.00	159.58	164.61	167.63
14	91	155.05	8.41	1.3177	155.8299	0.0526	142.16	145.22	150.27	155.83	161.32	166.22	169.12
15	110	157.35	8.32	1.9529	157.2717	0.0517	143.31	146.50	151.69	157.27	162.67	167.38	170.14
16	93	157.73	7.48	2.8349	157.7877	0.0510	143.36	146.78	152.17	157.79	163.06	167.55	170.13
17	100	156.56	8.90	3.8636	157.5656	0.0507	142.49	146.22	151.90	157.57	162.71	166.96	169.37

n, number of measurements; SD, standard deviation.

**Table 2:** Average, standard deviation (SD), L, M, S, and smoothed weight percentiles in male and female children and adolescents with intellectual disabilities aged between 7 and 17 years.

		Male (weight, kg)												
Age (years)	n	Average		SD	L	M	S	P5	P10	P25	P50	P75	P90	P95
7	50	27.37	7.32		−0.7294	26.0109	0.2675	17.76	19.15	21.96	26.01	31.56	38.59	44.22
8	60	31.51	9.06		−0.6711	29.5938	0.2786	19.85	21.49	24.79	29.59	36.18	44.51	51.17
9	93	34.30	10.63		−0.6054	33.4563	0.2888	22.04	23.96	27.83	33.46	41.16	50.87	58.57
10	97	44.33	15.99		−0.5223	37.6673	0.2966	24.39	26.63	31.14	37.67	46.53	57.54	66.14
11	123	43.60	14.71		−0.4210	41.4374	0.3004	26.45	29.00	34.12	41.44	51.21	63.06	72.10
12	114	47.82	14.75		−0.3160	45.5565	0.2999	28.81	31.70	37.45	45.56	56.15	68.62	77.88
13	131	52.02	15.28		−0.2185	50.2270	0.2959	31.63	34.89	41.31	50.23	61.60	74.62	84.03
14	157	59.03	18.75		−0.1304	54.9563	0.2894	34.63	38.26	45.32	54.96	66.97	80.37	89.83
15	140	61.78	17.89		−0.0517	58.9408	0.2803	37.37	41.29	48.83	58.94	71.27	84.70	93.99
16	126	64.54	16.58		0.0210	62.0283	0.2699	39.71	43.83	51.69	62.03	74.39	87.55	96.50
17	137	65.73	17.55		0.0907	64.5061	0.2593	41.75	46.03	54.08	64.51	76.73	89.49	98.03

		Female (weight, kg)												
Age (years)	n	Average		SD	L	M	S	P5	P10	P25	P50	P75	P90	P95

7	21	27.89	9.02		−0.0005	26.5873	0.2889	16.53	18.36	21.88	26.59	32.31	38.50	42.77
8	44	32.09	9.87		−0.0209	30.8410	0.2898	19.19	21.30	25.38	30.84	37.51	44.77	49.79
9	46	38.34	12.44		−0.0431	35.0336	0.2906	21.83	24.21	28.82	35.03	42.66	51.00	56.79
10	63	39.05	10.51		−0.0718	39.1857	0.2915	24.46	27.10	32.24	39.19	47.77	57.22	63.83
11	69	46.17	13.14		−0.1131	44.0374	0.2924	27.57	30.51	36.23	44.04	53.76	64.58	72.21
12	82	51.70	14.75		−0.1571	48.9117	0.2935	30.71	33.94	40.25	48.91	59.81	72.07	80.80
13	72	55.99	17.23		−0.1905	53.0114	0.2946	33.35	36.81	43.62	53.01	64.92	78.44	88.15
14	91	57.95	16.91		−0.2027	56.1197	0.2959	35.28	38.94	46.15	56.12	68.80	83.27	93.69
15	110	63.08	20.44		−0.1923	58.1749	0.2973	36.46	40.28	47.78	58.17	71.38	86.41	97.23
16	92	61.87	18.71		−0.1530	58.7676	0.2987	36.59	40.51	48.19	58.77	72.12	87.19	97.95
17	100	60.91	20.01		−0.0972	58.4191	0.3002	36.06	40.04	47.80	58.42	71.68	86.47	96.90

n, number of measurements; SD, standard deviation.

### Smoothed percentiles

**Figure 1:**
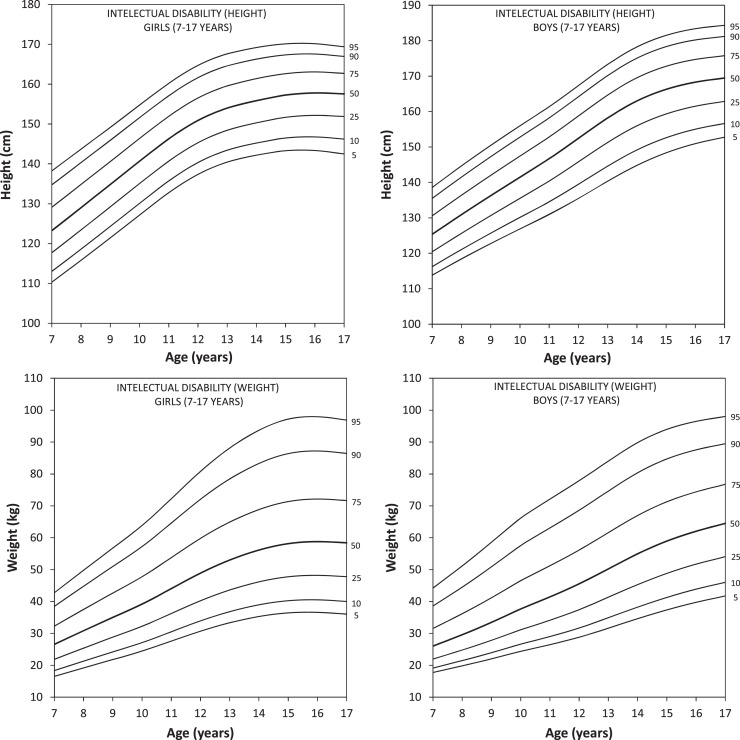
Percentiles (5, 10, 25, 50, 75, 90, 95) for height (panels A and B) and weight (panels C and D) in children and adolescents with intellectual disability by sex between ages 7 and 17 years old.

The percentiles for height and weight (P5, P10, P25, P50, P75, P90 and P95) were developed for children and adolescents of both sexes, aged between 7 and 17 years (Figure 1A – D; Table 1, Table 2), with satisfactory LMS modeling based on Q tests (Q statistic between −2 and +2) (Supplementary Fig. 2A – 2D) and worm plots (free from linear trend) (Supplementary Fig. 3A – 3D).

Boys with ID showed a linear trend in height up to 11 or 12 years old, followed by an increment from 13 to 15 years old and deceleration from 15 or 16 years old. Girls with ID showed a linear trend in height between 7 and 11 years old and a deceleration from the age of 12 years. Furthermore, girls with ID did not show substantial changes in height from 15 to 17 years of age.

Regarding weight, the percentiles developed in this study indicated that girls showed a linear tendency to gain weight up to 13 years of age, followed by a deceleration at 14 or 15 years of age and stabilization after 15 years of age, while boys showed a linear trend of weight gain from 7 to 17 years old.

## Discussion

This study developed smoothed height-to-age and weight-to-age percentiles, specifically for Brazilian children and adolescents with ID, aged between 7 and 17 years. The creation of percentiles has important implications for monitoring growth in the population with ID.

In this study, the height percentiles of boys with ID were linear until the age of 11 or 12, followed by an increment from the ages of 13 to 15 and a deceleration from the age of 15 or 16. Girls with ID showed a linear trend in height between the ages of 7 and 11 years. The data from this study are consistent with previous studies, indicating a linear trajectory of similar height between the sexes at ages below 11 years and substantially different after 12 years.^25^ Furthermore, girls with ID did not show substantial gains in height between the ages of 15 and 17. The results of growth rates during adolescence are in line with previous studies.^25^ The accelerated growth phase during puberty occurs two years earlier in girls compared to boys. From the age of 16, girls show minimal changes in height, while boys continue to grow substantially for another two or three years.^25^

Regarding weight, girls showed a linear weight trend until the age of 13, deceleration at the age of 14 or 15 and stabilization after the age of 15. However, boys showed a linear tendency to gain weight from 7 to 17 years of age. The findings of the present study are partially in agreement with findings about the population of children and adolescents without ID. Specifically, the present findings agree with those of Malina et al.,^25^ indicating linearity of weight in both sexes, from 7 to 12 or 13 years old, linearity up to 17 years old, in boys, and deceleration from 13 years old, in girls. However, Malina et al. data showed that girls continue to gain weight from the age of 15, while the girls with ID did not show weight gains from the age of 15.^25^

The development of percentiles can be useful in clinical practice, as is the case for subpopulations with ID. For example, previous studies developed height and weight percentiles for young people with Down,^11^^,^^21^ Williams,^13^ Rett,^14^ Rubinstein-Taybi,^15^ Prader-Willi,^16^ Wolf-Hirschhorn^26^ and Ellis–van Creveld^27^ syndromes, recommended by health agencies such as the CDC.^21^ The percentiles are a reference for health professionals to verify if there are deviations in weight or height or both between one appointment and the next and then they can establish a hypothesis to be evaluated. For example: if the patient changes to a higher percentile for weight and height, it could just be excess weight; if the patient changes to a higher percentile for weight and lower percentile for height, it could be hypothyroidism or Cushing disease.

However, evidence indicates that young people with ID associated with genetic conditions are shorter in height compared to young people with ID in the general population,^10^ which is a limitation for the clinical use of existing charts.

The present study is the first to develop growth references for height for people with ID not associated with genetic conditions. However, further studies are needed to confirm the multiethnic, age and sex variabilities in children with ID.

Sex and age are important determinants of health in the general population, but their effects on height are not entirely known. In young people with ID, research shows conflicting results regarding general health and lifestyle among boys and girls,^6^^,^^28^, ^29^, ^30^ limiting possible explanations for sex differences in height and weight in this population.

The present study has limitations. First, data was obtained retrospectively from clinical records. However, the data was obtained by trained professionals using standardized protocols. Furthermore, the data was rigorously subjected to cleaning processes. Secondly, the samples consisted of children and adolescents aged between 7 and 17 years, limiting the identification of critical age windows of growth, especially before 7 years of age. Thirdly, the sample size was relatively small in girls aged 7 to 9 years. However, diagnostic models used in the present study (i.e., Q statistics) considered sampling weights and presented a normal distribution, regardless of age. Lastly, although this study is the first to develop height and weight percentiles, its application in clinical practice should be examined in future studies.

This study developed height and weight percentiles with satisfactory modeling in boys and girls with ID, aged between 7 and 17 years. More research is needed to investigate possible changes in growth and variability with sex and age in children and adolescents with ID.

## Funding sources

The study was supported by the São Paulo 10.13039/100005930Research Foundation (FAPESP grants: 2017/13071-4; 2019/07103-6) and the São Paulo State Federation of APAES (FEAPAES-SP).

## Conflicts of interest

The authors declare no conflicts of interest.

## Appendix Supplementary materials

Image, application 1
