# Supplementary material for: Height and weight reference charts for Brazilians with intellectual disabilities aged 7–17
Source: J Pediatr (Rio J). 2024 Dec 4;101(2):269–75. doi: 10.1016/j.jped.2024.11.004 (PMC11889685; doi:10.1016/j.jped.2024.11.004)
Supplement: Supplementary file 1 [file mmc1.docx]

**JPED-D-24-00430**

**SUPPLEMENTARY MATERIALS**

**Figure 2** Q statistic for weight (panels A and B) and height (panels C and D) in children and adolescents with intellectual disabilities.

**Figure 3** *Detrended Q-Q plot (worm plot)* for weight (panels A and B) and height (panels C and D) in children and adolescents with intellectual disability.
